# Ahmed Glaucoma Valve Implantation for Refractory Glaucoma in a Tertiary Hospital in Brazil

**DOI:** 10.1155/2015/850785

**Published:** 2015-05-31

**Authors:** Ricardo Yuji Abe, Carla Melo Tavares, Rui Barroso Schimiti, José Paulo Cabral Vasconcellos, Vital Paulino Costa

**Affiliations:** ^1^Department of Ophthalmology, University of Campinas, Caixa Postal 6111, Campinas, SP, Brazil; ^2^Hospital de Olhos de Londrina, 86015-430 Londrina, PR, Brazil

## Abstract

*Purpose*. To evaluate the efficacy of Ahmed Glaucoma Valve (AGV) implantation in patients with refractory glaucoma in a tertiary hospital in Brazil. *Methods*. Retrospective case series of patients who underwent AGV implantation. Primary outcome was to assess the rate of failure, which was defined as intraocular pressure (IOP) in two consecutive visits greater than 18 or lower than 5 mmHg (criterion 1) or IOP greater than 15 or lower than 5 mmHg (criterion 2). The secondary outcome was to investigate risk factors for failure. *Results*. 112 eyes from 108 patients underwent AGV implantation between 2000 and 2012. Mean follow-up time was 2.54 (±1.52) years. Kaplan-Meier survival analysis showed cumulative probabilities of success of 80.3%, 68.2%, and 47.3% at 1, 3, and 5 years using 18 mmHg as endpoint. When adopting 15 mmHg as endpoint, cumulative success rates were 80.3%, 60.7%, and 27.3% at 1, 3, and 5 years, respectively. Multivariate analysis with generalized estimating equations revealed that African American ancestry and early hypertensive phase were risk factors for failure (*P* = 0.001 and *P* = 0.002, resp.). *Conclusion*. A success rate of approximately 50% was obtained 5 years after the implantation of an AGV. African American ancestry and early hypertensive phase were associated with increased risk of failure.

## 1. Introduction

Glaucoma drainage devices are typically reserved for refractory glaucomas, which are associated with high risk for failure, such as neovascular or uveitic glaucoma or after failed trabeculectomies [1, 2]. Although trabeculectomy still remains as the first-line surgical treatment for glaucoma, the use of drainage devices is becoming more common. Previous comparative studies were not able to demonstrate the superiority of trabeculectomy but reported fewer early postoperative complications when performing a glaucoma drainage device implantation [3, 4].

Ramulu et al. reported an increase from 2728 drainage device surgeries in 1995 to 7744 in 2004 according to Medicare data in the United States [5]. However, cost-effectiveness analysis and long-term follow-up are needed to evaluate if glaucoma drainage device can be used as primary surgical option. The Ahmed Glaucoma Valve (AGV) (New World Medical, Cucamonga, CA) and the Baerveldt glaucoma drainage implant (Abbott Medical Optics, Abbott Park, IL) are the two most commonly used glaucoma drainage devices [6, 7]. The AGV has a flow restrictor designed to reduce postoperative hypotony.

The “Ahmed Baerveldt Comparison Study” showed cumulative probabilities of failure of 31.3% and 44.7% after 3 and 5 years, respectively, following an AGV surgery [7, 8]. On the other hand, the “Ahmed versus Baerveldt Study” showed cumulative probability of failure of 51% after 3 years of AGV implantation [6]. There is no previous report of long-term follow-up of AGV surgeries in Brazil. The aim of the study is to analyze the survival rates of AGV implantation in patients with refractory glaucoma in a tertiary hospital in Brazil and identify possible risk factors for failure.

## 2. Methods

### 2.1. Patient Selection

This was a retrospective study, evaluating patients with refractory glaucoma who underwent an AGV implantation at Hospital das Clinicas, University of Campinas, Brazil. “Refractory glaucoma” was defined as glaucomas associated with a poor surgical prognosis after trabeculectomy, which remained uncontrolled despite previous filtration surgery and/or laser treatment and/or under maximum tolerated medical treatment. Only patients with at least 1 year of follow-up were included in the study, unless failure occurred before that period. All procedures were performed between January 2000 and November 2012. This study was approved by the Ethics Committee of the University of Campinas and adhered to the tenets of the Declaration of Helsinki. Medical records were retrospectively reviewed for preoperative and postoperative follow-up information, such as occurrence of complications. At each visit during follow-up, subjects underwent a comprehensive ophthalmic examination, including Snellen best corrected visual acuity, slit-lamp biomicroscopy, intraocular pressure (IOP) measurement using Goldmann applanation tonometry, gonioscopy, and dilated fundoscopy examination using a 78-diopter lens. Flat anterior chambers were graded according to the classification proposed by Spaeth [9].

### 2.2. Primary and Secondary Outcome Measures

The primary outcome measure of this study was to establish the rates of failure, which was defined by two consecutive visits with IOP greater than 18 mmHg or lower than 5 mmHg (criterion 1) or IOP greater than 15 mmHg or lower than 5 mmHg (criterion 2) after 3 months of surgery. Failure was also defined as the need of an additional glaucoma surgery for IOP control or loss of light perception during follow-up. Severe complications such as tube or plate exposure in which the implant had to be removed were also considered as failure. The secondary outcome of the study was to identify possible risk factors for failure following AGV surgery.

### 2.3. Surgical Technique and Postoperative Follow-Up

Surgeries were performed by experienced attending glaucoma surgeons (JPV, RBC, and VPC) or by glaucoma fellows under their direct surgical supervision. The AGV models used were the S2 polypropylene (184 mm^2^ surface area) and FP7 silicone (184 mm^2^ surface area). All surgeries were performed with peribulbar anaesthesia. A fornix-based conjunctival flap was fashioned preferably in the superotemporal quadrant. AGV was primed by flushing balanced salt solution through the tube to confirm patency. The anterior edge of the plate was secured with 9-0 nylon sutures to the sclera at least 8 mm from the limbus. A 23-gauge needle was used 1 mm posterior to the limbus to create a track and access the anterior chamber. A rectangular donor scleral patch graft (4 × 6 mm) was fashioned and sutured over the tube using 10-0 nylon sutures. The conjunctiva was also secured with 10-0 nylon sutures. No antimetabolites were used in the procedure. Follow-up visits were scheduled 1 day, 3 days, 1 week, 2 weeks, and 1, 3, 6, 12, and 18 months; after that, patients were followed within a 6-month interval. All patients received a standard regimen of topical antibiotic drops (moxifloxacin hydrochloride) q.i.d, discontinued after 2 weeks. Topical corticosteroids drops (prednisolone acetate ophthalmic suspension 1%) were used initially 6 times daily and tapered gradually over 6 to 10 weeks depending on the degree of inflammation. Glaucoma medications were prescribed according to IOP measurements and the severity of the disease. An early hypertensive phase (EHP) was defined as an IOP increase of more than 21 mmHg within the first 3 months after surgery and after a reduction of IOP to less than 21 mmHg had been achieved during the first postoperative week [10, 11].

### 2.4. Statistical Analysis

Snellen visual acuity and the semiquantitative scale “counting fingers,” “hand motion,” and “light perception” were converted to logarithm of the minimum angle of resolution (logMAR) for analysis [12]. Cumulative survival rates were calculated using Kaplan-Meier survival analysis. We included variables previously listed as risk factors according to [10, 13, 14]. Since both eyes of some patients were evaluated, univariate and multivariate analyses were performed with generalized estimating equations with the exponentiation results presented in Odds Ratios. Statistical analysis and artwork were performed using Stata, version 13 (StataCorp LP, College Station, Texas, USA). The alpha level (type I error) was set at 0.05.

## 3. Results

The present study included 112 eyes from 108 glaucoma patients who underwent an AGV implant.  Table 1 shows clinical and demographic characteristics of the subjects. Patients were followed for an average of 2.54 ± 1.52 years. Mean age was 59.73 ± 17.65 years. Among the 112 eyes, 58 received the Ahmed S2 valve (51.79%), and 54 eyes (48.21%) underwent Ahmed FP7 valve implantation. A total of 71 eyes (63.39%) had filtering surgery prior to the AGV implantation. The most common type of glaucoma was primary open angle glaucoma (POAG) (50.89%), followed by neovascular (20.54%), traumatic (6.09%), congenital (5.36%), and uveitic glaucoma (4.46%). This distribution is similar to previous studies such as Ahmed Baerveldt Comparison Study and the Ahmed Versus Baerveldt Study [6, 8]. Baseline average and postoperative logMAR visual acuity were 1.13 ± 0.68 and 1.23 ± 0.64, respectively (*P* = 0.021) (Table 2). Mean IOP before surgery was 29.00 ± 9.72 mmHg and 16.69 ± 7.08 mmHg at the last follow-up (*P* < 0.001). Mean number of medications decreased from 3.50 ± 0.67 prior to surgery to 2.44 ± 1.27 at the last follow-up (*P* < 0.001) (Table 2).

Kaplan-Meier analysis utilizing criterion 1 (5 mmHg < IOP < 18 mmHg) showed cumulative survival rates of 80.3%, 68.2%, and 47.3%, after 1, 3, and 5 years, respectively. When criterion 2 was employed (5 mmHg < IOP < 15 mmHg), cumulative survival rates of 80.3%, 60.7%, and 27.3% were observed after 1, 3, and 5 years, respectively. The most common reason for failure was reintervention due to high IOP levels (69.2%) (Table 3). Among the 39 failures, 2 patients (5.1%) had severe complications and 6 patients (15.3%) lost light perception during follow-up. We observed three (1.6%) intraoperative complications (hyphema, lens touch, and malignant glaucoma) (Table 4). The most common early complication was choroidal detachment (6 cases, 5.0%), which occurred after a mean of 4.4 days after surgery, followed by shallow anterior chamber grades 1 and 2 (5 cases, 4.2%), occurring after a mean of 3 days after surgery (Table 4). The most common late complication was implant exposure (5 cases, 4.2%), after a mean of 10.2 months (Table 4).

**Figure 1:**
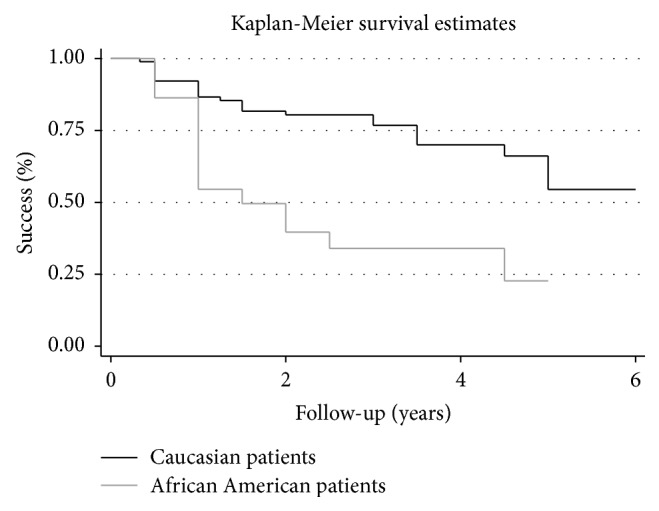
Percentage of success after Ahmed Glaucoma Valve implantation in Caucasian and African American patients, using failure definition of intraocular pressure greater than 18 mmHg and less than 5 mmHg. Univariate analysis showed that African American ancestry was a risk factor for failure (*P* = 0.001).

**Figure 2:**
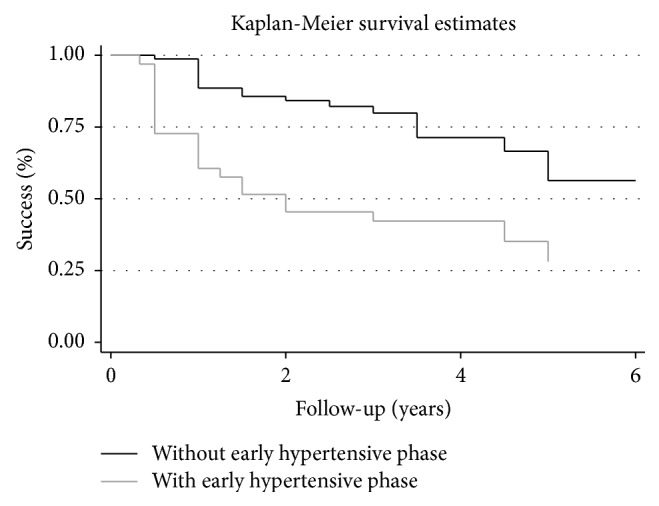
Percentage of success after Ahmed Glaucoma Valve implantation in patients with and without the occurrence of the early hypertensive phase during follow-up, using failure definition of intraocular pressure greater than 18 mmHg and less than 5 mmHg. Univariate analysis showed that early hypertensive phase was a risk factor for failure (*P* < 0.001).

Univariate analysis (based on criterion 1) showed that African American (Figure 1) descent and EHP (Figure 2) were risk factors for failure (*P* = 0.001; *P* < 0.001, resp.) (Table 5). Other covariates such as age, gender, previous glaucoma filtering surgery, the type of the implant used, and other types of glaucoma were not significantly associated with failure. On the multivariate analysis (based on criterion 1), African-American descent and EHP remained as risk factors for failure (*P* = 0.001 and *P* = 0.002, resp.). African American patients had a risk of 5.51 (CI, confidence interval) (CI: 2.32–19.06) and patients with EHP had a risk of 4.88 (CI: 1.74–11.60) for AGV failure (Table 5).

We also performed a separate analysis to study the effects of EHP on the African American and Caucasian patients. Univariate analysis (based on criterion 1) showed that, in the African American group, EHP was not considered a risk factor for failure (*P* = 0.605), whereas in the Caucasian group, EHP had odds ratio of 7.36 (CI: 2.62–12.62; *P* < 0.001).

## 4. Discussion

The present study disclosed a cumulative success rate of 80.3% at 1 year, 68.2% at 3 years and 47.3% at 5 years using 18 mmHg as endpoint. For a stricter endpoint (15 mmHg), cumulative success rates were 80.3%, 60.7% and 27.3% at 1, 3 and 5 years, respectively. In the current study, the cumulative success rate was different from the results of the “Ahmed Baerveldt Comparison Study” (cumulative probabilities of success of 68.7% and 55.3% after 3 and 5 years, resp.), because they defined failure as IOP more than 21 mmHg [8]. However, our results were similar to the “Ahmed Versus Baerveldt Study” (with cumulative probability of success of 49% after 3 years), which used the same of IOP levels (5 to 18 mmHg) as in our study [6]. Although not common in the literature, adopting a stricter criterion (15 mmHg) for the definition of surgical success is important due to the fact that some patients with advanced glaucoma need lower IOP levels to avoid progression of the disease.

The secondary outcome of this study was to identify possible risk factors for failure following AGV implantation. According to the multivariate analysis, African American descent was significantly associated with risk of failure. This finding is in agreement with a previous study from Ishida and Netland, where 86 eyes from 43 African Americans and 43 Caucasian patients were followed for an average of 2.5 years after AGV implantation [14]. In their study, two different definitions for success were adopted. The first was based on IOP levels between 6 and 21 mmHg with or without the use of additional glaucoma medications. The second definition required the same IOP levels and also a 20% IOP reduction from baseline. Despite not finding statistically significant differences regarding the number of complications and mean IOPs during follow-up, the authors reported that African American ancestry was a risk factor for surgical failure for both definitions (*P* = 0.030 and *P* = 0.006, resp.).

The pathophysiological basis underlying racial differences on AGV implant survival is unknown. Several studies confirmed that African American ancestry is characterized by high prevalence and rapid progression of glaucoma [15, 16]. Additionally, medical therapy seems to be less effective in African Americans when compared to Caucasians [17]. Finally, there is evidence suggesting a higher failure rate for trabeculectomy in African American patients [18].

Following AGV implantation, a fibrotic response encapsulates the plate of the drainage device, leading to increased aqueous outflow resistance [19, 20]. Histopathological analysis from the inner layer of the encapsulated bleb showed transformation of fibroblasts into myofibroblasts in the collagen fibers. This process is regulated by cytokines, such as the transforming growth factor-*β* (TGF-*β*) [20, 21], which mediate the fibrotic process that occurs in patients undergoing glaucoma filtration and drainage implant surgery [22]. Freedman and Iserovich found higher levels of inflammatory cytokines in the aqueous from patients with encysted blebs from Molteno implants [23]. In addition, Trivedi et al. found a trend toward higher aqueous humor concentrations levels of TGF-*β* in African American subjects, suggesting that racial differences on inflammatory cytokines levels might explain differences in failure rates following AGV implant surgeries [24].

Our study also found that the hypertensive phase was a risk factor for AGV failure. In our sample, the hypertensive phase occurred in 33 from the total of 112 eyes (29.4%), among which 21 failed (63.6%). Previous studies have reported an incidence of EHP ranging from 56 to 82% following the implantation of a glaucoma drainage device [10, 25]. The pathophysiological basis underlying the occurrence of the EHP might be explained by fibroblasts activating an inflammatory reaction as part of the healing process in the scar formation, resulting in fibrosis and poor functioning of the drainage device [26]. In addition, early contact of aqueous inflammatory mediators with the conjunctiva and Tenon's capsule may induce cell death followed by collagen fiber swelling, fragmentation, and opening of the intercellular matrix [26]. This wound healing process and scar formation around the implant create a fibrotic capsule that might make the aqueous drainage difficult, increasing IOP levels [27].

The material used in glaucoma drainage implants can also play role in fibrous tissue formation and encapsulation [20]. In fact, Ishida et al. evaluated 132 patients in a prospective comparative series between silicone (FP7) and polypropylene (S2) plates [28]. The study found improved success after implantation of FP7 device compared with S2 AGV. In addition, a higher incidence of Tenon's cyst formation requiring treatment was observed in the S2 group. Therefore, polypropylene plate material and the occurrence of a hypertensive phase were considered as risk factors associated with failure [28]. However, in our study there was no difference in survival rates between the S2 and the FP7 models of the AGV implants (Table 5).

Two recent prospective clinical trials have investigated the adoption of lower IOP targets during follow-up by initiating early aqueous suppressants treatment following AGV implantation [11, 29]. Pakravan et al. evaluated 94 patients who underwent AGV implantation to study the effects of early initiation of aqueous suppressant treatment during postoperative follow-up. These patients were divided into two different groups, in which the limit for IOP was 10 mmHg and 15 mmHg, respectively. They found significantly lower IOP, a higher success rate, and less frequent hypertensive phases in eyes that adopted a lower IOP target (10 mmHg). The overall success rate in this group after 1 year of follow-up was 63.2%, compared to 33.3% in the other group (*P* = 0.008) [11]. Law et al. evaluated 52 eyes undergoing AGV implantation that were randomized to initiate aqueous suppressant therapy when postoperative IOP was higher than 10 mmHg in one group or higher than 17 mmHg in the other group, during follow-up [29]. They found that humor aqueous suppression in the early postoperative period while IOPs were still in the low-teens was able to reduce the incidence of IOP spikes associated with the hypertensive phase without increasing the complications rate.

We performed a separate analysis within the Caucasian and African American groups to better investigate the relationship between EHP and race. Among 14 African American patients without EHP, 9 (64.2%) failed. Among the 8 African Americans in which EHP occurred, 6 patients (75.0%) failed. In the Caucasian group, 11 (16.9%) of the 65 patients without EHP failed and 15 (60.0%) from 25 in which EHP occurred failed. These findings suggest that, in the African American group, EHP does not represent a risk factor for failure. In fact, in the univariate analysis, EHP was not considered a risk factor in the African American patients (*P* = 0.605). On the other hand, EHP had odds ratio for failure of 7.36 (CI: 2.62–12.62; *P* < 0.001) in the Caucasian group, suggesting that lowering IOP with early use of aqueous suppressants might not be as effective in African Americans compared to Caucasians. Future studies should evaluate prospectively the impact of racial differences on the early aqueous suppressants treatment and the onset of EHP.

Our study is limited by its retrospective nature and the absence of a control arm to compare the results of the AGV implantation. Although different surgeons were responsible for the AGV implantation, all surgeries were performed under direct surgical supervision of experienced attending glaucoma specialists and followed a standardized technique. This study was conducted in a public hospital, where the cost of the AGV implant limits its widespread use. Hence, it is possible that the sole inclusion of the refractory glaucomas may have reduced the survival rates of the implant. Also the indication for new glaucoma procedures was based on subjective clinical judgment; however, only two patients who underwent surgical reinterventions had IOPs lower than 18 mmHg.

In conclusion, AGV implantation is associated with a reasonable success rate in refractory glaucoma, reaching 47.3% after 5 years. However, significant differences on survival rates were observed when adopting a stricter IOP endpoint (≤15 mmHg). African American descendants and patients who present EHP in the postoperative period are at greater risk for AGV implantation failure.

## Figures and Tables

**Table 1:** Demographic and clinical characteristics of study patients.

Parameters	Total subjects (*n* = 108)
Follow-up (years)	
Mean ± SD	2.54 ± 1.52
Range	0.33 to 6.00
Age (years)	
Mean ± SD	59.73 ± 17.65
Range	14 to 86
Gender, *n* (%)	
Male	58 (53.70%)
Female	50 (46.30%)
Race, *n* (%)	
Caucasian	84 (77.78%)
African American	21 (19.44%)
Others	2 (2.87%)
Comorbidity, *n* (%)	
Hypertension	27 (37.50%)
Diabetes	24 (33.33%)

Parameters	Total eyes (*n* = 112)

Eye, *n* (%)	
Right	60 (53.57%)
Left	52 (46.43%)
Previous glaucoma surgery, *n* (%)	
Yes	71 (63.39%)
Type of implant, *n* (%)	
Ahmed S2	58 (51.79%)
Ahmed FP7	54 (48.21%)
Type of glaucoma, *n* (%)	
Primary open angle	57 (50.89%)
Neovascular	23 (20.54%)
Traumatic	7 (6.09%)
Congenital	6 (5.36%)
Uveitic	5 (4.46%)
Penetrating keratoplasty	5 (4.46%)
Primary angle closure	2 (1.79%)
Others	7 (6.09%)

SD: standard deviation; S2: polypropylene; FP7: silicone.

**Table 2:** Comparison between baseline and postoperative visual acuity, intraocular pressure, and glaucoma medications.

Parameters	Baseline	Postoperative	*P* value
Visual acuity (log⁡MAR)			
Mean ± SD	1.13 ± 0.68	1.23 ± 0.64	0.021
IOP			
Mean ± SD	29.00 ± 9.72	16.69 ± 7.08	<0.001
Glaucoma medications			
Mean ± SD	3.50 ± 0.67	2.44 ± 1.27	<0.001

SD: standard deviation; IOP: intraocular pressure.

**Table 3:** Reasons for treatment failure after Ahmed Glaucoma Valve surgery.

Reason for failure	*N* (%)
18 mmHg < IOP < 5 mmHg and reintervention for IOP control	27 (69.2%)
18 mmHg < IOP < 5 mmHg or	2 (5.12%)
Reintervention for IOP control	2 (5.12%)
Severe complications	2 (5.12%)
Loss of light perception	6 (15.3%)

IOP: intraocular pressure.

**Table 4:** Intraoperatory, early and late complications related to Ahmed Glaucoma Valve surgery.

Intraoperatory complications		
Complication	Number of cases	Time
Hyphema	1	N/A
Lens touch	1	N/A
Malignant glaucoma	1	N/A

Early complications		
Complication	Number of cases	Time in days (mean)

Choroidal detachment	6	4.2
Shallow anterior chamber grades 1 and 2	5	3.3
Shallow anterior chamber grade 3	4	5.75
Hypotony maculopathy	3	6.6
Hyphema	2	1
Corneal edema	2	1
Endophthalmitis	1	3
Tube-corneal touch	1	3
Tube exposure	1	7

Late complications		
Complication	Number of cases	Time in months (mean)

Tube exposure	5	10.2
Corneal edema	4	2
Phthisis bulbi	2	3
Tube extrusion	2	16.5
Vitreous hemorrhage	2	18.5
Retinal detachment	2	4.5
Cystoid macular edema	1	3
Macular hole	1	6
Strabismus	1	6
Plate exposure	1	12
Hyphema	1	12
Uveitis	1	24

**Table 5:** Univariate and multivariate analysis to evaluate risk factors following Ahmed Glaucoma Valve surgery.

Risk factors	Univariate		Multivariate		
Parameters	OR	*P* value	OR	95% CI	*P* value
Age (y)					
≥60 years	0.98	0.087	0.78	0.28–2.12	0.631
<60 years					
Gender					
Male	1.3	0.504	1.21	0.46–3.21	0.693
Female					
Race					
White	5.27	0.001	5.51	2.32–19.06	0.001
African American					
Previous glaucoma surgery					
Yes	1.01	0.997	1.74	0.44–6.79	0.423
No					
Type of glaucoma					
Primary open angle	0.46	0.068	0.99	0.26–4.91	0.989
Neovascular glaucoma	1.43	0.444	3.18	0.59–16.80	0.229
Uveitic	7.56	0.075	6.86	0.90–11.10	0.060
Traumatic	1.78	0.489	NA	NA	NA
Congenital	0.33	0.319	NA	NA	NA
Primary angle closure	1.73	0.587	NA	NA	NA
Penetrating keratoplasty	0.41	0.443	NA	NA	NA
Early hypertensive phase					
Yes	5.15	<0.001	4.88	1.74–11.60	0.002
No					
Type of implant					
Ahmed S2	0.55	0.141	0.54	0.20–1.46	0.229
Ahmed FP7					

IOP: intraocular pressure, OR: odds ratio, CI: confidence interval, and NA: not applicable.

S2: polypropylene; FP7: silicone.

All analyses were performed using generalized estimating equation.

## References

[1] Papadaki T. G., Zacharopoulos I. P., Pasquale L. R., Christen W. B., Netland P. A., Foster C. S. (2007). Long-term results of Ahmed glaucoma valve implantation for uveitic glaucoma. *The American Journal of Ophthalmology*.

[2] Arcieri E. S., Paula J. S., Jorge R. (2015). Efficacy and safety of intravitreal bevacizumab in eyes with neovascular glaucoma undergoing ahmed glaucoma valve implantation: 2-year follow-up. *Acta Ophthalmologica*.

[3] Minckler D. S., Vedula S. S., Li T. J., Mathew M. C., Ayyala R. S., Francis B. A. (2006). Aqueous shunts for glaucoma. *Cochrane Database of Systematic Reviews*.

[4] Gedde S. J., Herndon L. W., Brandt J. D., Budenz D. L., Feuer W. J., Schiffman J. C. (2012). Postoperative complications in the tube versus trabeculectomy (TVT) study during five years of follow-up. *American Journal of Ophthalmology*.

[5] Ramulu P. Y., Corcoran K. J., Corcoran S. L., Robin A. L. (2007). Utilization of various glaucoma surgeries and procedures in Medicare beneficiaries from 1995 to 2004. *Ophthalmology*.

[6] Christakis P. G., Tsai J. C., Kalenak J. W. (2013). The ahmed versus baerveldt study: three-year treatment outcomes. *Ophthalmology*.

[7] Barton K., Feuer W. J., Budenz D. L. (2014). Three-year treatment outcomes in the ahmed baerveldt comparison study. *Ophthalmology*.

[8] Budenz D. L., Barton K., Gedde S. J. (2015). Five-year treatment outcomes in the ahmed baerveldt comparison study. *Ophthalmology*.

[9] Spaeth G. L. (1990). Glaucoma surgery. *Ophthalmic Surgery: Principles & Practice*.

[10] Nouri-Mahdavi K., Caprioli J. (2003). Evaluation of the hypertensive phase after insertion of the Ahmed Glaucoma Valve. *American Journal of Ophthalmology*.

[11] Pakravan M., Rad S. S., Yazdani S., Ghahari E., Yaseri M. (2014). Effect of early treatment with aqueous suppressants on Ahmed glaucoma valve implantation outcomes. *Ophthalmology*.

[12] Schulze-Bonsel K., Feltgen N., Burau H., Hansen L., Bach M. (2006). Visual acuities ‘hand motion’ and ‘counting fingers’ can be quantified with the Freiburg Visual Acuity Test. *Investigative Ophthalmology & Visual Science*.

[13] Li Z., Zhou M. W., Wang W. (2014). A prospective comparative study on neovascular glaucoma and nonneovascular refractory glaucoma following ahmed glaucoma valve implantation. *Chinese Medical Journal*.

[14] Ishida K., Netland P. A. (2006). Ahmed glaucoma valve implantation in African American and white patients. *Archives of Ophthalmology*.

[15] Wilson R., Richardson T. M., Hertzmark E., Grant W. M. (1985). Race as a risk factor for progressive glaucomatous damage. *Annals of Ophthalmology*.

[16] Leske M. C., Wu S.-Y., Hennis A., Honkanen R., Nemesure B. (2008). Risk factors for incident open-angle glaucoma: the Barbados Eye Studies. *Ophthalmology*.

[17] Netland P. A., Robertson S. M., Sullivan E. K. (2003). Response to travoprost in black and nonblack patients with open-angle glaucoma or ocular hypertension. *Advances in Therapy*.

[18] Morris D. A., Peracha M. O., Shin D. H., Kirn C., Cha S. C., Kim Y. (1999). Risk factors for early filtration failure requiring suture release after primary glaucoma triple procedure with adjunctive mitomycin. *Archives of Ophthalmology*.

[19] Lee J. W., Park W. Y., Kim E. A., Yun I. H. (2014). Tissue response to implanted Ahmed glaucoma valve with adjunctive amniotic membrane in rabbit eyes. *Ophthalmic Research*.

[20] Eibschitz-Tsimhoni M., Schertzer R. M., Musch D. C., Moroi S. E. (2005). Incidence and management of encapsulated cysts following Ahmed glaucoma valve insertion. *Journal of Glaucoma*.

[21] Tomasek J. J., Gabbiani G., Hinz B., Chaponnier C., Brown R. A. (2002). Myofibroblasts and mechano: regulation of connective tissue remodelling. *Nature Reviews Molecular Cell Biology*.

[22] Grehn F., Hollo G., Khaw P. (2007). Factors affecting the outcome of trabeculectomy: an analysis based on combined data from two phase III studies of an antibody to transforming growth factor beta2, CAT-152. *Ophthalmology*.

[23] Freedman J., Iserovich P. (2013). Pro-inflammatory cytokines in glaucomatous aqueous and encysted Molteno implant blebs and their relationship to pressure. *Investigative Ophthalmology & Visual Science*.

[24] Trivedi R. H., Nutaitis M., Vroman D., Crosson C. E. (2011). Influence of race and age on aqueous humor levels of transforming growth factor-beta 2 in glaucomatous and nonglaucomatous eyes. *Journal of Ocular Pharmacology and Therapeutics*.

[25] Ayyala R. S., Zurakowski D., Smith J. A. (1998). A clinical study of the Ahmed glaucoma valve implant in advanced glaucoma. *Ophthalmology*.

[26] Bae K., Suh W., Kee C. (2012). Comparative study of encapsulated blebs following Ahmed glaucoma valve implantation and trabeculectomy with mitomycin-C. *Korean Journal of Ophthalmology*.

[27] Rosbach J., Choritz L., Pfeiffer N., Thieme H. (2013). Clinical results of encapsulated bleb removal after Ahmed glaucoma valve implants. *Der Ophthalmologe*.

[28] Ishida K., Netland P. A., Costa V. P., Shiroma L., Khan B., Ahmed I. I. K. (2006). Comparison of polypropylene and silicone Ahmed Glaucoma Valves. *Ophthalmology*.

[29] Law S. K., Kornmann H. L., Giaconi J. A. (2014). Early aqueous suppressant therapy on hypertensive phase following glaucoma drainage device procedure: a randomized prospective trial. *Journal of Glaucoma*.

